# Validation of the interpersonal needs questionnaire of young male adults in Singapore

**DOI:** 10.1371/journal.pone.0198839

**Published:** 2018-06-07

**Authors:** Dorothy C. H. Teo, Lidia Suárez, Tian P. S. Oei

**Affiliations:** 1 Department of Psychology, James Cook University, Singapore; 2 School of Psychology, The University of Queensland, Brisbane, Australia; Universitat Wien, AUSTRIA

## Abstract

Interpersonal needs are associated with suicide. However, no interpersonal needs questionnaire has been validated in Asia. The psychometric properties of the Interpersonal Needs Questionnaire were examined in a sample of 340 young male adults in Singapore. This questionnaire measures proximal causes of desire for suicide using the extent to which individuals believe that they are a burden to others (perceived burdensomeness) and the extent to which they believe their needs are not met (thwarted belongingness). Confirmatory factor analysis found that a two-factor model of perceived burdensomeness and thwarted belongingness provided an adequate fit for the data. Internal consistency was excellent. Concurrent, convergent, predictive, and discriminant validity was demonstrated. The findings showed that the Interpersonal Needs Questionnaire provides a valid measure of interpersonal needs in young Asian males. Thus, the instrument can be utilized to test the interpersonal psychological theory of suicidal behavior.

## Introduction

Suicide is a salient problem with similar rates around the world. For instance, the suicide prevalence rate for young people is 10.2 per 100,000 individuals in the US, 11.5 in Japan, and 8.7 in Singapore [1]. The suicide process is a progression starting from suicidal ideation, plans about taking one’s life, through suicide acts with increasing lethality and suicidal intent [2]. It is crucial to examine and measure risk factors for suicidal ideation.

A parsimonious theory of the extenuating contexts underlying suicidal ideation is the interpersonal-psychological theory of suicidal behavior [3], which posits that two key interpersonal factors are perceived burdensomeness (PB) and thwarted belongingness (TB). PB refers to the belief that one is a burden to significant others, and that they, therefore, would be better off if one were dead. TB refers to the belief that there is lack of care or concern from others, as well as lack of meaningful connections with others. When these two needs are unmet, there is psychological maladjustment, such as passive suicidal ideation [3]; and when there is hopelessness too, hopelessness predicts active suicidal ideation [4]. Other factors associated with suicidal ideation are depression, lack of meaning in life, lack of social support, and deficits in problem-solving appraisal [5–8].

The Interpersonal Needs Questionnaire (INQ) was created to measure the constructs of PB and TB [9], but only a few studies have employed it in Asian populations and, to the extent of our knowledge, no study has explored its psychometric properties in Asia. Zhang et al. [8] administrated an 18-item version of the INQ in China, and reported high internal consistency (Cronbach’s alpha = .87). In the US, Wong et al. [10] administrated the INQ-18 to a sample of Asian-American students. They reported internal consistencies of .74 for PB and .88 for TB, and found that PB was significantly and positively associated with suicidal ideation. The INQ-18 has also shown good internal consistency (.86 for PB and .84 for TB) in more ethnically heterogeneous populations in the US, but the two-factor structure of the INQ-18 could not be validated [11]. Zaroff et al. [12] employed the INQ-12 in Macau and found adequate internal consistency for PB (Cronbach’s alpha = .75) but low internal consistency for TB (Cronbach’s alpha = .43). In contrast, the internal consistency of the INQ-12 has been found to be adequate in the US [11,13], with Cronbach’s alphas between .87 and .93 for PB, and .80 to .92 for TB, but the INQ-12 showed poor factorial validity [11]. To summarize, the INQ-18 has demonstrated good internal consistency when used in China and the US. The INQ-12 has shown poor internal consistency when used with a Macau (China) sample but strong internal consistency in US samples. Moreover, the structural validity of the INQ-18 and INQ-12 does not fit the two-factor model hypothesized by the interpersonal-psychological theory of suicidal ideation [3], at least in North-American samples.

In contrast to the INQ-18 and INQ-12, the INQ-15 has shown good internal consistency and factorial validity, as well as concurrent and convergent validity in US populations [9, 11]. The current study tested the psychometric properties of the INQ-15 in Asia, in particular, Singapore. This is in line with the position held by Van Orden et al. [9], who suggested that there is a need for research on the INQ measurement model using samples of “increased diversity in terms of race/ethnicity” and that “before the INQ is used in other countries, the content validity of the INQ items for the specified culture should be examined.” (p. 212).

The lack of studies examining the construct validity of the INQ in Asia, and the finding regarding the low internal consistency of the TB scale in China call into question the construct validity of this subscale and invites exploration of its validity in Asia. Examining its factorial validity in an Asian context is a first step in testing the interpersonal psychological model [3]. It cannot be assumed that the latent concepts of PB and TB, which were conceptualized and tested on US samples, will be equivalent in Asian contexts. People in Western societies predominantly adopt an independent self-construal, characterized by the self as an autonomous entity and having attributes that drive the individual’s motives, behaviors and emotions. On the other hand, people in Asian societies are more likely to adopt an interdependent self-construal, whereby others are incorporated into one’s sense of self [14–17]. In Western societies, others are less centrally placed in one’s self-identity, whereas in Asian societies, there is a strong emphasis on family cohesion, self-restraint, and group harmony [18]. PB and TB might be an especially critical cultural risk factor for suicidal desire in Asians because of the importance of harmonious interpersonal relationships and group cohesion in their lives [10].

In the present study, it was hypothesized that the INQ-15 would fit a two-factor model (representing the PB and TB constructs) and have good internal consistency. It was also predicted a positive correlation between interpersonal needs, depression and problem-solving appraisal, and a negative correlation between interpersonal needs and hope. Additionally, good convergent validity was expected by finding high shared variance among the items forming each of the two factors of the model. Moreover, it was hypothesized that the scores in the INQ-15 would predict suicidal desire to a greater extent than suicidal plans. Discriminant validity was tested by demonstrating that the constructs of PB and TB are unique and different from the relationship between the two constructs.

## Method

### Participants

From the 400 survey packages that were mailed, 360 were returned, and 342 were usable. Packages that were returned and discarded contained missing data. The final sample included 342 young males living in Singapore and aged between 18 to 24 years (*M* = 20.41, *SD* = 1.32). All the participants were English speakers. The sample was a non-clinical sample. Participants had to have at least secondary school education to rule out difficulties in reading. The ethnic background of the sample was Chinese (69%), Malay (17%), Indian (5.6%) and other races (8.4%).

The current study focused on young males with the purpose of examining the validity of measures of risks factors of suicidal ideation in Asia, and specifically in those serving or about to serve National Service, which is compulsory for males in Singapore. Military service involves separation from family members, and elevated levels of loneliness and thwarted belongingness have been found among military personnel [19]. Military service is a common problem reported by young people who attempt suicide [20], and there is a rapid rise in the military suicide rate [21]. Moreover, males are less likely to manifest the early warnings of suicide than females [22], but are more likely to die from suicide [19,23–25].

### Measures

Demographic data were collected, and the following self-report instruments were used.

#### Interpersonal needs questionnaire (INQ) [9]

The INQ-15 is a self-report questionnaire used to measure the two constructs of PB and TB. It is comprised of two subscales. The first six statements measure PB (e.g., “These days, I think I am a burden on society”), and the last nine statements measure TB (e.g., “These days, I feel like I belong”). Participants were asked to indicate the degree to which each item had been true for them recently. A 7-point Likert scale was used, ranging from “not at all true for me” to “very true for me”. Six items on the TB scale were reverse scored. Higher scores reflected higher levels of PB and TB. The INQ-15 subscales have demonstrated good internal consistency, with Cronbach’s alpha values ranging from .85 to .90 for PB, and .81 to .87 for TB [11,26].

#### Depression Anxiety Stress Scales-D scale (DASS-D) [27]

The DASS-21 is a self-report measure comprised of 21 items that measure self-reported levels of emotional distress. The DASS-21 is comprised of three subscales, namely depression, anxiety and stress. In this study, the 7-item DASS Depression subscale was used, comprising items 3, 5, 10, 13, 16, 17, and 21. This scale assesses depressive symptoms, such as dysphoria, self-deprecation and anhedonia (e.g., “I felt I had nothing to look forward to”). Participants were asked to rate the extent to which they had experienced depressed state over the past week using a 4-point Likert scale ranging from “did not apply to me at all” to “applied to me very much or most of the time”. Higher scores indicated greater levels of depressed state. The DASS-D has good internal consistency, with Cronbach’s alpha values ranging between .81 and .88 across Asian samples [28].

#### Hope Scale [29]

The Hope Scale was used to measure overall perception that aspirations can be met. It is comprised of 12 items (four are fillers). Using a 4-point Likert scale that ranged from “definitely false” to “definitely true”, respondents were asked to describe the extent that each sentence applied to them. Higher scores suggested higher levels of hope. In this scale, hope was represented by two subscales, the Agency and Pathway subscales. Agency describes people’s sense of successful determination in relation to their goals generally, and the subscale was comprised of items 2, 9, 10 and 12. An item example is, “I meet the goals that I set for myself”. Pathway describes people’s cognitive appraisals of their ability to generate means of overcoming goal-related obstacles and reaching their goals. This subscale was comprised of items 1, 4, 6 and 8. An item example is “There are lots of ways around any problem”. The scale has been found to have good internal consistency, with Cronbach’s alphas ranging from .71 to .76 for the Agency subscale, and .63 to .80 for the Pathway subscale [29].

#### Suicide Ideation Scale (SIS) [30]

The SIS is a 10-item questionnaire used to evaluate suicide risk. It assesses passive suicidal desire (e.g., “I feel life isn’t worth living”, items 5 to 8) and active suicidal planning (e.g., “I have been thinking of ways to kill myself”, items 1 to 4, 9, and 10). It uses 5-point Likert scales, with scores ranging from “never” to “always”. Higher scores indicate higher levels of suicidal ideation. The subscales have been found to have good internal consistency (Cronbach’s alpha = .89 for passive suicidal desire, and .89 for active suicidal planning) [6].

#### Problem Solving Inventory (PSI) [31]

The PSI is a self-report inventory which assesses respondents’ appraisal of their problem-solving capacity. Respondents rated their degree of agreement to statements that described how they reacted to personal problems in their day-to-day life on a 5-point Likert scale. Responses ranged from “strongly agree” to “strongly disagree”. Lower scores indicated more successful problem solving. There were 35 items of which three items were filler items. Reverse scoring was required on 14 of the items. Lower scores indicated more successful problem-solving appraisal. The PSI comprises three subscales: problem-solving confidence (PSC–items 5, 10, 11, 12, 19, 23, 24, 27, 33, 34 and 35), approach-avoidance style (AAS–items 1, 2, 4, 6, 7, 8, 13, 15, 16, 17, 18, 20, 21, 28, 30, 31) and personal control (PC–items 3, 14, 25, 26 and 32). Item examples include, “I have the ability to solve most problems even though initially no solution is immediately apparent”, which measured PSC; “I try to predict the result of a particular course of action”, which measured AAS; and “I make snap judgments and later regret them”, which measured PC. The PSI has acceptable internal consistency, with average Cronbach’s alpha coefficients in the low-to-mid .80s for PSC and AAS, and in the low .70s for PC [32].

### Procedure

Subjects were recruited from a university in Singapore and the community. They participated for credit points or as volunteers. They were informed about the study through advertisement flyers and snow-ball recruitment. Persons interested in participating contacted the researchers through email or in person. Upon request, a survey package was either sent by mail or given to the participants in person.

The survey package contained a cover sheet outlining the research and instructions, the informed consent form, the questionnaires, a mental health tips leaflet and information on avenues for help, and a self-addressed pre-paid reply envelope. The cover page contained information about voluntary participation, anonymity, instructions on how to return the survey package and information on the importance of maintaining mental well-being. Participants were informed that their responses would be anonymous and that responding to the questions could potentially be distressing. They were encouraged to read the mental health leaflet, which outlined tips on what they could do and venues for help if they felt distressed. Respondents completed the survey on their own time. Responses were returned by post in the self-addressed pre-paid sealed envelope provided.

The Human Research Ethics Committee of James Cook University approved this study (Approval number H5062).

## Results

### Data screening prior to analysis

Two multivariate outliers were identified and were removed from the database. Thus, the final sample size was 340. Multivariate distribution of the INQ was found to be non-normal. As a result, bootstrapping was used by generating 5000 subsamples from the original database, which generated the 90% bias-corrected confidence interval for the factor loading estimates. There were no issues of linearity or collinearity among the measures employed in this study.

### CFA

AMOS version 21 was used to perform two CFAs with maximum likelihood estimation. The first CFA tested a one-factor model of general distress, in which the 15 items loaded on a single dimension, as in Freedenthal et al.’s study [13]. The second CFA tested the hypothesis that the INQ represented the PB and TB factors and that they were related to each other, as illustrated in the interpersonal-psychological theory of suicidal behavior [3]. Multiple measures were used to assess goodness-of-fit, such as χ^2^, the standardized squared root mean squared residual (SRMR), root mean squared error of approximation (RMSEA), comparative fit index (CFI), and Tucker-Lewis index (TLI). SRMR and RMSEA values less than .08, and CFI and TLI values equal to or more than .90 were considered adequate.

The data did not fit a one-factor model of general distress, χ^2^(90) = 1219.23, *p* < .001, SRMR = .13, RMSEA = .19, CFI = .72, TLI = .67. The results, however, indicated that the data fit a two-factor model, χ^2^(88) = 373.24, *p* < .001, SRMR = .07, RMSEA = .10, CFI = .93, TLI = .92, after an error covariance between two items corresponding to the TB factor was estimated, as in Van Orden et al.’s study [9]. Table 1 presents the CFA two-factor model and standardized factor loadings, all *p* < .001. Factor loadings were large for PB and moderate for TB. As theorized, there was a positive correlation between the two latent constructs (*r* = .66, *p* < .001). The results of the present study were similar to those of the bootstrap sample, suggesting that the results may be replicable in a normal sample.

**Table 1:** INQ -15 Model estimated unstandardized factor loadings (+SE), standardized factor loadings, R^2^ values, and item-total correlations.

	Unstand. Estimate	*SE*	Stand. Estimate	*R*^*2*^	Item-total Correlation
Perceived Burdensomeness (PB)^a^					
Better off	.96	.04	.90	.80	.85
Happier without me	.99	.04	.92	.85	.89
Burden to society	1.05	.05	.88	.77	.86
Death as a relief	.88	.04	.89	80	.87
Rid of me	.89	.05	.85	.72	.83
Makes worse	1.00	-	.84	.70	.83
Thwarted Belongingness (TB)^a^					
Others care	.84	.07	.63	.70	.53
I belong	1.09	.07	.81	.66	.75
Rarely interact	.59	.08	.44	.20	.46
Friends	1.00	.07	.77	.59	.72
Disconnected	.81	.08	.57	.32	.63
Outsider	.89	.08	.61	.37	.66
Turn to	1.06	.07	.78	.61	.69
Close to others	1.11	.07	.82	.67	.76
Daily interact	1.00	-	.74	.55	.68
Covariances					
PB with TB	.97	.12	.66	-	-
Disconnected with Outsider	1.44	.15	.70	-	-

^a^The last item of each latent variable was set to 1.00.

### Internal consistency

Medium and large item-total correlations indicated that items in the two factors represented the constructs of PB and TB adequately (Table 1). The Cronbach’s alpha values for the INQ and the other scales used in this study are presented in Table 2. All the scales had good internal consistency.

**Table 2:** Descriptives, internal reliability and bivarate correlations between DASS-D, hope, PSI, SIS, and INQ scores.

				Correlation		
	*M*	*SD*	Cronbach’s Alpha	Total INQ	PB	TB
Depression^a^	.72	.75	.92	.74	.70	.65
Hope^b^	2.97	.50	.86	-.58	-.48	-.56
Problem-Solving Appraisal^c^	2.88	.60	.91	.50	.44	.46
Suicidal Ideation^c^	1.29	.60	.95	.69	.75	.55
Passive Suicidal Desire^d^	1.37	.76	.94	.71	.75	.57
Active Suicidal Planning^d^	1.24	.54	.92	.62	.69	.48
INQ^e^	2.46	1.10	.93	-	.86	.94
Perceived Burdensomeness^e^	1.89	1.18	.95	-	-	.64
Thwarted Belongingness^e^	2.84	1.24	.89	-	-	-

^a^Scores obtained with the DASS-D

^b^scores obtained with the Hope Scale

^c^scores obtained with the PSI

^d^scores obtained with the SIS

^e^scores obtained with the INQ. All correlations *p* < .001.

### Concurrent validity

In line with the interpersonal-psychological theory of suicidal behavior [3], it was found that PB, TB and the overall INQ were positively associated with depression and negatively associated with hope (see Table 2). Depression and the INQ shared 54.76% of variance, and hope and the INQ shared 33.64% of the variance. The INQ also correlated with problem-solving appraisal (shared variance = 25%), indicating that the more interpersonal problems, the more problem-solving appraisal issues.

### Convergent validity

The average variance extracted (AVE) of PB (.77) and TB (.49) indicated that the items in each of the two constructs shared a fair proportion of variance, confirming convergent validity.

### Predictive validity

It was hypothesized that INQ responses would predict suicidal desire, and (to a lesser degree) suicidal plans. Simple regression analyses showed that the scores of the INQ predicted suicidal desire, R^2^ = .50, *F*(1, 338) = 333.39, *p* < .001; and suicidal plans, R^2^ = .38, *F*(1, 338) = 209.94.39, *p* < .001. As expected, INQ predicted suicidal desire to a greater extent than suicidal plans.

### Discriminant validity

Discriminant validity was shown by higher squared root AVEs values for PB (.89) and TB (.70) than the correlation between them (.66). The results indicated that the constructs of PB and TB were distinct from each other.

## Discussion

The INQ-15 showed good construct validity, internal consistency, concurrent validity, convergent validity, predictive validity, and discriminant validity in a Singapore male young adult sample.

CFA confirmed adequate fit for a two-factor model, which was comprised of PB and TB. The CFA values were similar to the ones obtained in previous research [9,11], although the RMSEA values obtained in this study were slightly above the cut-off criterion. Standardized factor loadings obtained in the current study mirrored the ones obtained in the US by Van Orden et al. [9], although the average of standardized factor loadings for PB were higher in this study (.88) than in Van Orden et al.’s study (.70). The two research teams obtained similar averages of standardized factor loadings for TB (current study = .69; Van order et al. = .65). The findings suggest that PB is a more robust construct than TB in Singapore than in the US, and that PB is more important than TB in suicide ideation, as shown by the correlations in Table 2. Wong et al. [10] also found that PB was more important than TB in predicting suicide ideation in Asians. The correlation between PB and TB obtained in this sample was .66, similar to the value of .67 obtained by Van Orden et al. [9]. It is worth noting that Van Orden et al.’s sample was composed of only 33% males, while the sample used in the present study was made up solely of males. Considering that previous research [9,11] generalized the two-factor model to males and females, older, and clinical populations in the US, and that the present researchers found that the model fitted an Asian male-only sample, the results suggest that the model could generalize to a wide range of different populations.

In line with the interpersonal-psychological theory of suicidal behavior [3], the results of the current study suggest that the INQ more strongly predicts desire for suicide than for suicidal planning. Furthermore, the findings demonstrated that the INQ was more associated to the constructs of depression (*r* = .74) than hope (*r* = -.58) and problem-solving appraisal (*r* = .50). Also, the findings are consistent with research showing that suicide risk is present in depression, and that problem-solving issues and hopelessness increase the risk [5,33]. In this study, it was found that PB and depression shared a large quantity of variance (*r*^2^ = .49), suggesting that depression symptoms are closely linked to PB. PB (*r* = .75) was more strongly associated with suicidal ideation than TB (*r* = .55), as in previous research conducted in Asia [8,10]. These findings have important clinical implications. To reduce the risk of suicide, therapy will need to focus on feelings of PB and depression, which might be exacerbated by life transitions and low controllability such as serving the army. Van Order et al. [9] found convergent validity between PB and autonomy, responsibility to family and self-competence. From a therapeutic perspective, dealing with issues related to autonomy, responsibility, and self-competence might decrease PB and the risk of suicidal ideation. Hopelessness and problem-solving appraisal are also important aspects to be considered during therapy. Problem-solving appraisal is associated with suicide ideation, and it is mediated by hopelessness [5]. When people feel they cannot cope with their problems, they can become hopeless about the future, and suicidal ideation might arise [5]. Therefore, therapeutic strategies designed to deal with problem-solving appraisal are critical to prevent hopelessness, which is a predictor of active suicidal ideation [4].

Limitations of this study are that it focuses on young adult males only, and the findings cannot be generalized across age groups, the female gender, or to clinical populations in Singapore. Future studies should aim to replicate the current findings in more heterogeneous samples across Asia.

In conclusion, the INQ has good psychometric properties and can be used to assess how young adult males living in the Singapore community perceive meeting interpersonal psychological needs. This is also the first step necessary to test the interpersonal-psychological theory of suicide in Singapore.
